# Performance of large language models on the radiation and cancer biology practice exam

**DOI:** 10.3389/fonc.2026.1738955

**Published:** 2026-05-19

**Authors:** Jessica Bertschmann, Yang Xu, Conrad Bayley, Ahmad Abdellatif, Sangjune Laurence Lee

**Affiliations:** 1 Division of Radiation Oncology, Arthur Child Cancer Centre, Calgary, AB, Canada; 2 Department of Electrical & Software Engineering, University of Calgary, Calgary, AB, Canada

**Keywords:** ChatGPT, large language models (LLMs), medical education, natural language processing (NLP), radiation biology

## Abstract

**Background/objectives:**

Large Language Models (LLMs) are increasingly used in medicine for tasks ranging from patient communication to exam preparation. This study aimed to evaluate the feasibility of using a domain-specific, out-of-training-data radiation and cancer biology examination as a benchmarking framework for large language models, and to compare the accuracy and consistency of commonly used LLMs available at the time of data collection.

**Methods:**

GPT-3.5, GPT-4, and Llama-2 were queried with 335 multiple-choice questions (MCQs) from the 2023 American Society for Radiation Oncology (ASTRO) Radiation and Cancer Biology Exam Study Guide, excluding image-based items. Each model answered all questions five times over three months to evaluate consistency. Model responses were scored against the official answer key and analyzed using one-way ANOVA with Bonferroni correction to determine statistical differences in accuracy.

**Results:**

GPT-4 achieved the highest accuracy, correctly answering 81% of questions, significantly outperforming GPT-3.5 (62%) and Llama-2 (51%) (p < 0.001). All models performed worse on questions requiring calculations, though differences were not statistically significant. In terms of reliability, GPT-4 and Llama-2 provided consistent responses more frequently than GPT-3.5. Despite stable overall scores, all models exhibited variability in individual responses across repeated trials. GPT-4 produced the longest explanations, averaging 183 words per answer.

**Conclusions:**

This study demonstrates the feasibility of using a domain-specific, out-of-training-data examination to benchmark large language model knowledge in radiation and cancer biology. While performance differences were observed among models, variability and limitations, particularly in calculation-based questions, highlight the importance of methodological benchmarking and cautious interpretation when considering medical educational applications.

## 1 Introduction

Large Language Models (LLMs) are advanced artificial intelligence systems designed to understand and generate human-like text through using deep learning algorithms. These models have been trained on large datasets, enabling them to perform a wide array of tasks ranging from simple question-answering to complex problem-solving in various domains and topics (1). Popular examples include ChatGPT by OpenAI, which used the Generative Pre-trained Transformer (GPT)-3.5 and GPT-4 models, and Large Language Model Meta AI (Llama)-2 by Meta.

These powerful models have attracted significant interest in various fields including healthcare and medical education (2). Notably, ChatGPT has shown promising performance by achieving passing scores on all steps of the United States Medical Licensing Exam (USMLE) (3). Multiple LLMs have since been tested on specialized medical board examinations across different disciplines, achieving results comparable to those of trained medical professionals (4–8). Fields such as radiation oncology, which require do-main-specific expertise beyond general medical knowledge and are highly dependent on a continuous influx of new clinical trial data and evolving guidelines, stand to gain immensely from such advancements (9). As such, there has been a recent surge of re-search effort to evaluate what impact LLMs have on various tasks in the field of radiation oncology. For instance, they have been assessed on their ability to answer patient questions (10, 11), assist in tumor board rounds (12, 13), and answer exam questions (14–17), with varied results.

Despite a growing body of literature evaluating large language model (LLM) performance on oncology-related examination questions, several important gaps remain. Many prior studies have relied on question sets that may overlap with model training data, limiting the ability to assess true knowledge generalization. In addition, most studies report single-pass accuracy without systematically evaluating response variability across repeated queries, an important consideration given the known non-deterministic behavior of LLMs (18). Furthermore, relatively few studies have focused specifically on foundational radiation and cancer biology, which underpins clinical decision-making in radiation oncology but represents a distinct knowledge domain compared to clinical oncology or patient-facing tasks.

To address these limitations, we evaluate the feasibility of a domain-specific, out-of-training-data examination as a standardized benchmarking framework. Using the 2023 American Society for Radiation Oncology (ASTRO) Radiation and Cancer Biology Study Guide, which is temporally outside the models’ training cutoff, we minimize data leakage and more rigorously assess knowledge. In addition to accuracy, we quantify response consistency across repeated trials to characterize intra-model variability. Accordingly, we assess the baseline accuracy and response consistency of three widely used LLMs on multiple-choice questions from this study guide under a standardized, closed-book, no-prompt setting, isolating intrinsic model knowledge and providing a reproducible benchmarking framework rather than replicating real-world use cases involving prompting or external retrieval.

## 2 Materials and methods

The 2023 ASTRO Radiation and Cancer Biology Exam Study Guide was used to evaluate the accuracy of GPT-3.5, GPT-4, and Llama-2. The multiple-choice questions were obtained from the official ASTRO website and are free and publicly available (19). Questions containing graphical or image-based content were excluded (2/337 questions excluded) as GPT-3.5 and Llama-2 did not support image input at the time of data collection.

The exam questions were classified into the ten foundational topics listed on ASTRO’s study guide, including: 1) Interaction of radiation with matter, 2) Molecular and cellular damage and repair, 3) Cellular response to radiation, 4) Linear energy transfer (LET) and oxygen effect, 5) Tumor biology and microenvironment, 6) Cancer biology, 7) Radiobiology of normal tissues, 8) Dose delivery, 9) Combined modality therapy, and 10) Late effects and radiation protection. Questions were also categorized based on whether they required the use of math.

The LLMs queried include OpenAI’s ChatGPT-3.5 and GPT-4, and Meta’s Llama-2. None of the LLMs had the ability to search the internet or external databases. All data were collected between August–December 2023. The exact backend model versions may evolve over time and our findings represent model performance at the defined timepoint.

At the time of data collection, the LLMs were trained on data up until September 2021, therefore the 2023 ASTRO exam represented out-of-training-data. For consistency, each MCQ question, including its lettered answer choices, was individually inputted into the designated text field of each LLM’s web interface with no additional text in the prompt. Each question was inputted individually in the order in which they were presented on the ASTRO sample exam, and all parts of each question were provided as they appeared on the exam. To reduce bias due to the retention of previous questions and answers, a new chat session was started for each question. The answer choice provided in the response from each LLM as well as their explanation was recorded. The answers generated by each LLM were compared to the answer key provided and scored as either correct or incorrect. Instances in which the LLM responded with “none of the above,” “insufficient information,” or “declined to answer due to ethical concerns” were marked as incorrect.

To assess the consistency of LLM responses, each model was presented with the same set of 335 questions five times over the span of three months. Changes in overall score as well as a breakdown of response changes among the five tests were recorded.

The one-way analysis of variance (ANOVA) was used to determine statistically significant differences between LLMs’ accuracy. In situations where the ANOVA indicated significant differences, pairwise analysis was conducted using the Bonferroni correction to adjust for multiple comparisons, with the significance level set at alpha <0.0167. All statistical evaluations were performed using the R statistical software version 4.2.1 (R Foundation for Statistical Computing, Vienna, Austria).

## 3 Results

### 3.1 Accuracy of large language models on radiation and cancer biology exam questions

**Figure 1:**
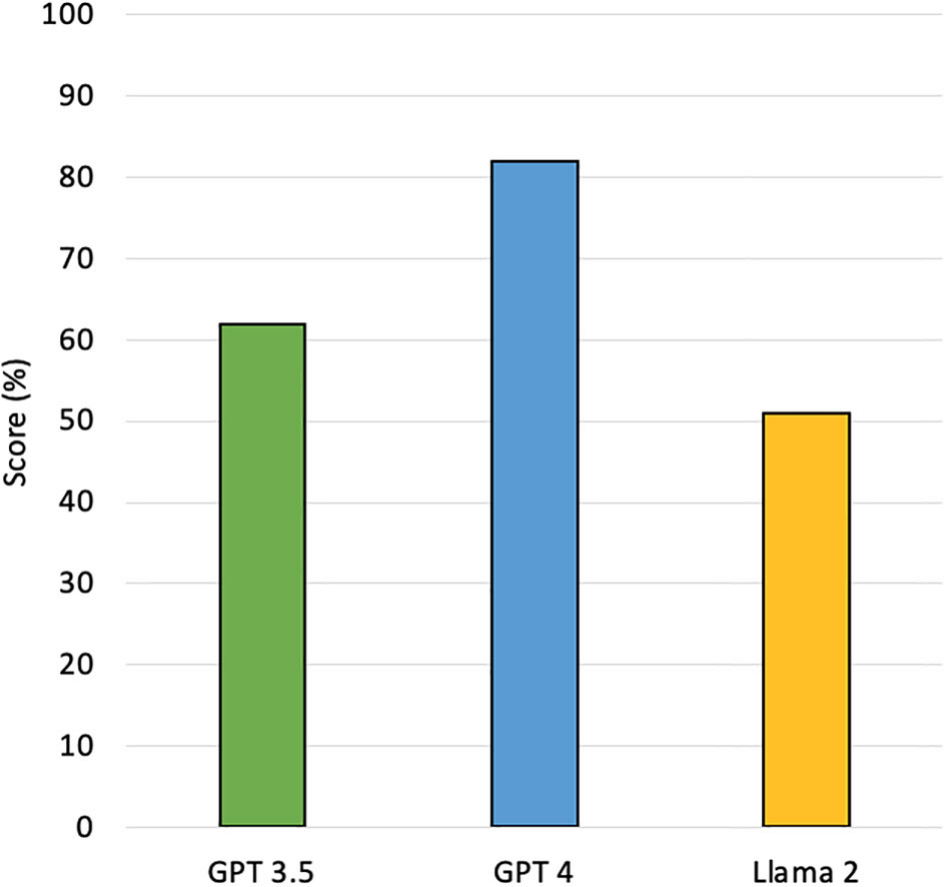
The overall accuracy of LLMs on the 2023 ASTRO radiation and cancer biology study guide on first examination attempt.

A total of 335 MCQ questions were queried by each LLM. GPT-3.5 scored 208/335 (62%), GPT-4 scored 272/335 (81%), and Llama-2 scored 173/335 (52%) on first examination attempt (**Figure 1**). In terms of overall accuracy, GPT-4 significantly outperformed GPT-3.5 by 19% (p < 0.001), and Llama-2 by 30% (p < 0.001). GPT-3.5 also significantly outperformed Llama-2 by 11% (p < 0.001).

**Figure 2:**
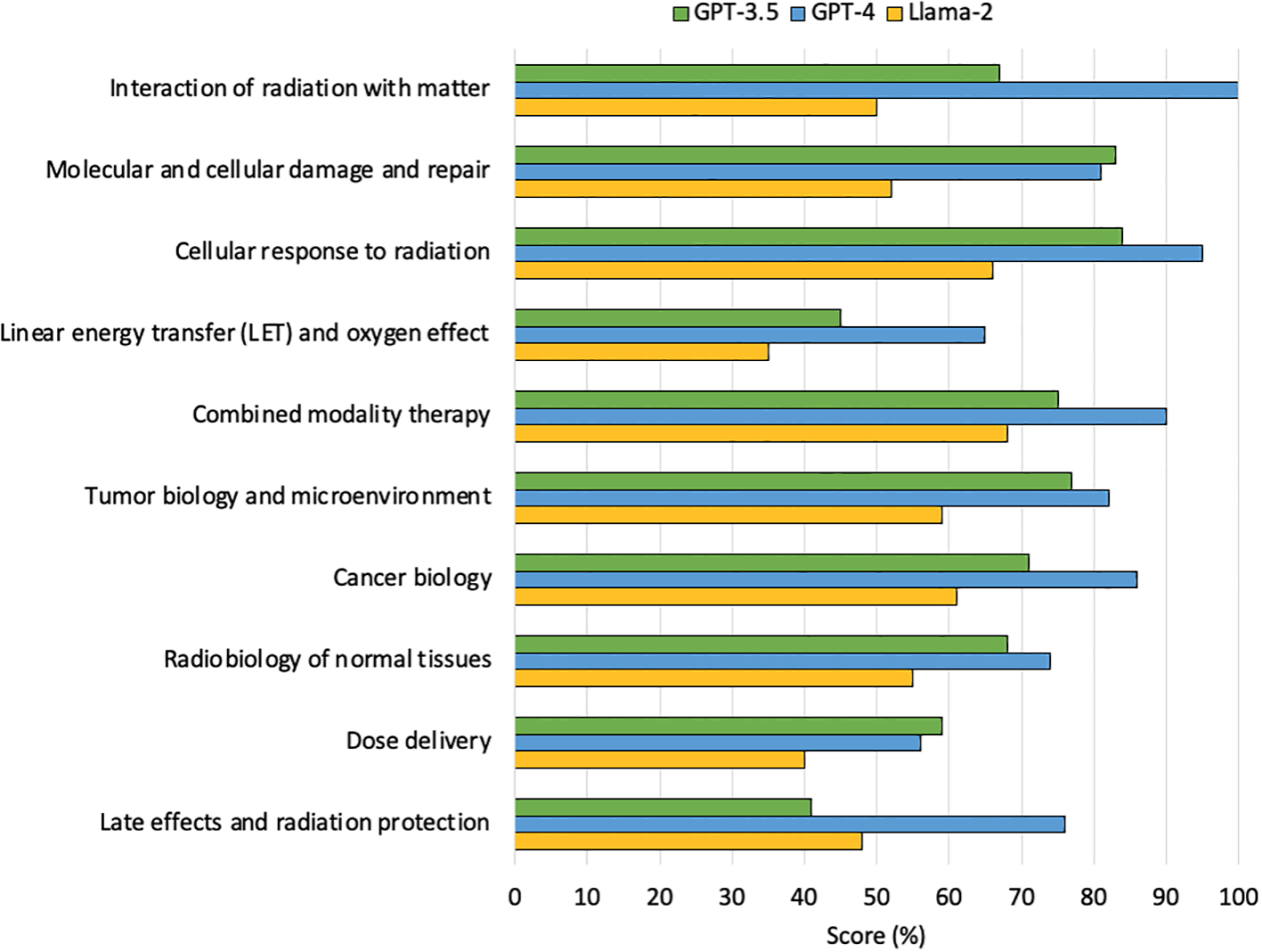
Percentage of questions answered correctly by the LLMs on each topic category on first examination attempt.

GPT-3.5 was most accurate on questions related to cellular response to radiation, scoring 37/44 (84%), and least accurate on question related to late effects and radiation protection, scoring 12/29 (41%). GPT-4 was most accurate on questions related to inter-action of radiation with matter, scoring 12/12 (100%) and least accurate on questions related to a dose delivery, scoring 18/32 (56%). Llama-2 was most accurate on questions related to combined modality therapy scoring 17/20 (68%) and least accurate on LET and oxygen effect scoring 7/20 (35%). Comprehensive results are depicted in **Figure 2**.

**Figure 3:**
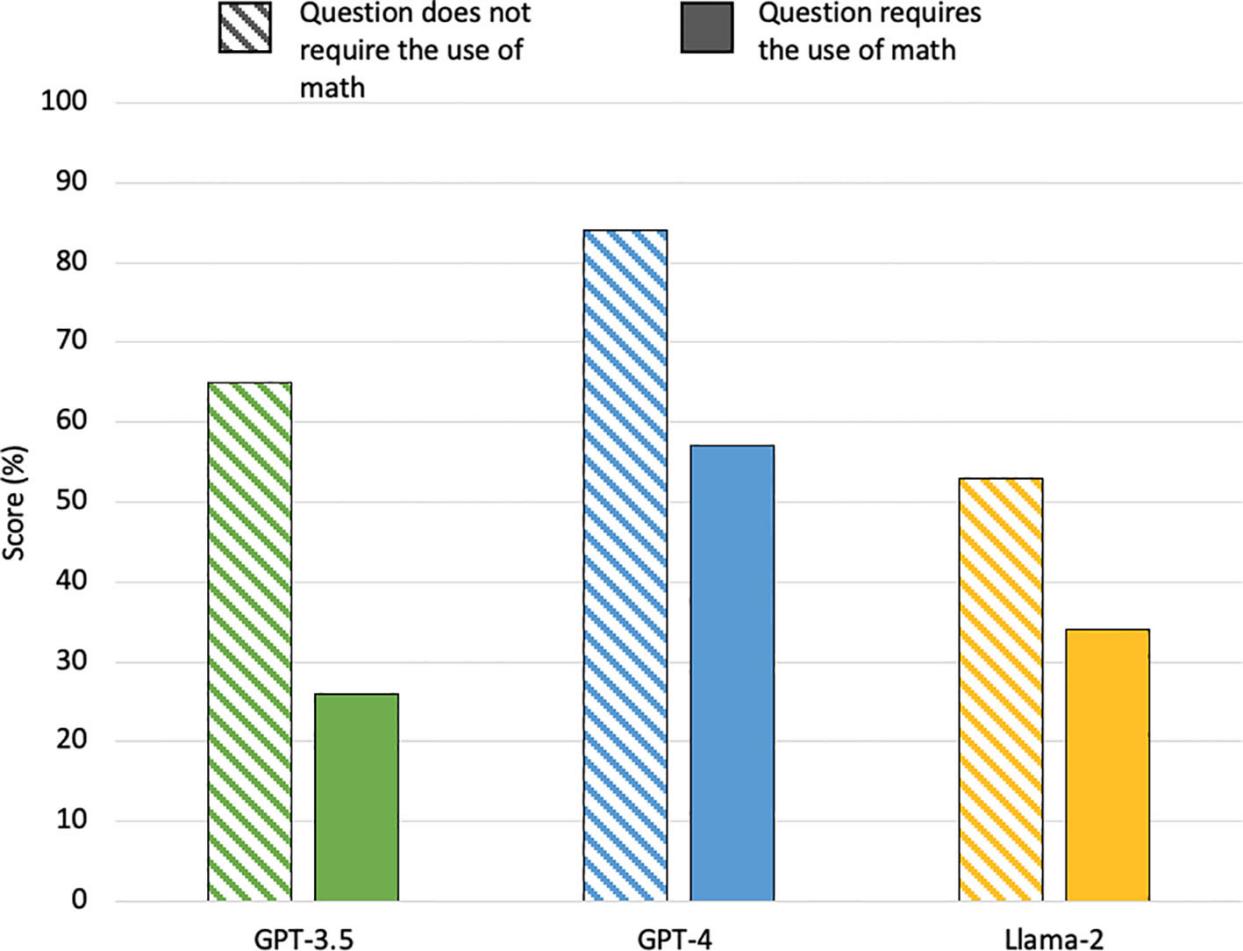
Percentage of questions answered correctly by the LLMs on questions requiring the use of math on first examination attempt.

All 3 LLMs had lower accuracy on questions requiring the use of calculations compared to questions that did not require calculations (**Figure 3**). Of the 26 questions requiring the use of calculations, GPT-3.5 scored 7/26 (26%), GPT-4 scored 15/26 (57%), and Llama-2 scored 9/26 (34%). Overall, GPT-4 outperformed GPT-3.5 by 31% and Llama-2 by 23%. Llama-2 also outperformed GPT-3.5 by 8%. These differences were not statistically significant.

### 3.2 Reliability of large language models on radiation and cancer biology exam questions

**Figure 4:**
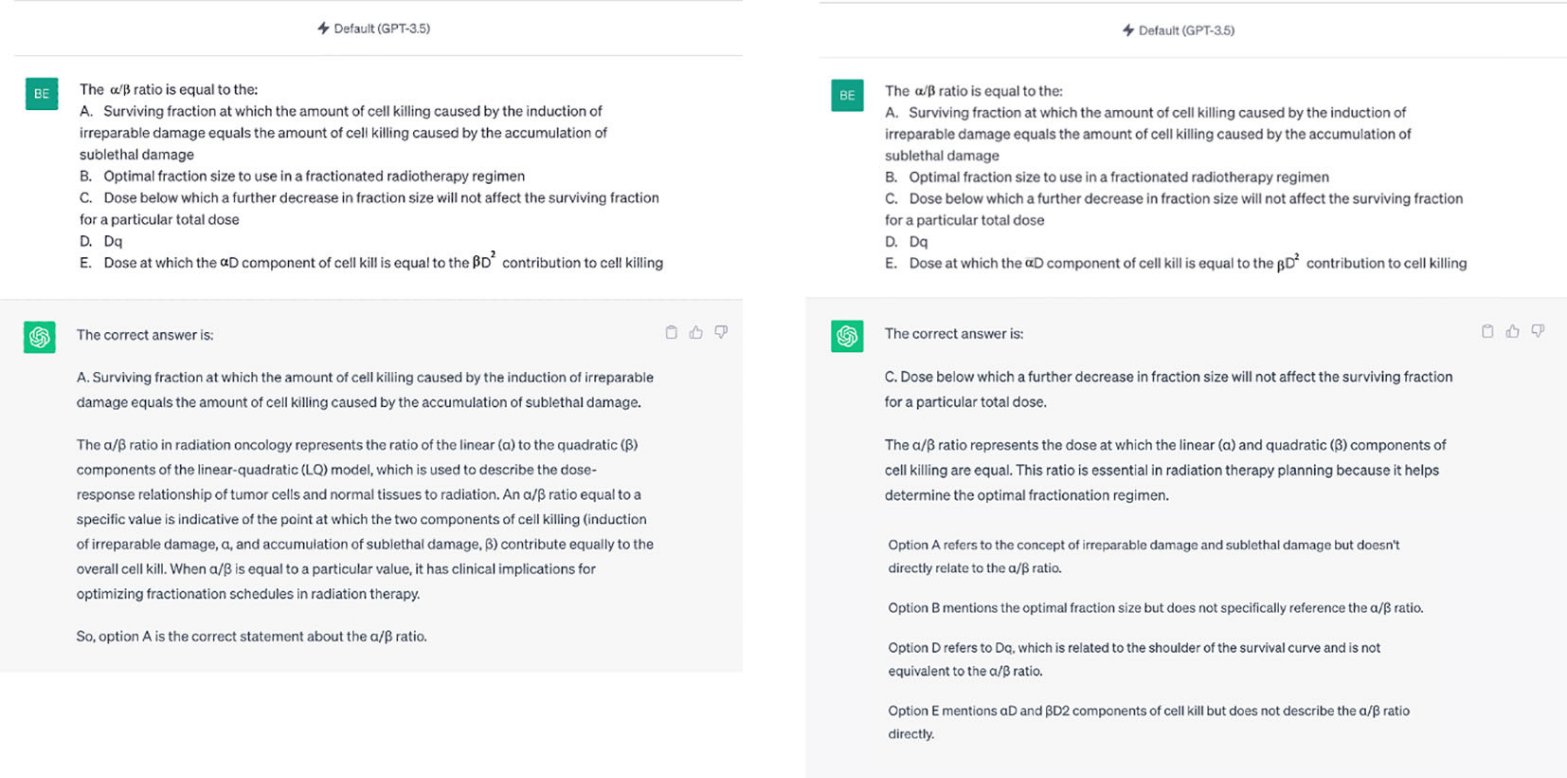
An example of two distinct outputs produced by GPT-3.5 in response to an identical input.

In the assessment of reliability of LLMs, overall test scores were largely consistent among the five evaluations (**Table 1**). Despite the relative stability in test scores, a closer examination revealed that all 3 LLMs would frequently generate different answers in response to identical questions. **Figure 4** shows an example of two distinct outputs produced by GPT 3.5 in response to the same input. Changes in overall scores due to different responses between each examination attempt are summarized in **Table 2**.

**Table 1:** The overall accuracy of LLMs on 5 separate attempts of the 2023 ASTRO radiation and cancer biology study guide.

Examination attempt	GPT-3.5	GPT-4	Llama-2
Exam attempt 1	208/335 (62%)	272/335 (81%)	173/335 (52%)
Exam attempt 2	201/335 (60%)	274/335 (82%)	171/335 (51%)
Exam attempt 3	205/335 (61%)	274/335 (82%)	180/335 (54%)
Exam attempt 4	202/335 (60%)	269/335 (80%)	173/335 (52%)
Exam attempt 5	206/335 (61%)	270/335 (81%)	176/335 (53%)

**Table 2:** Consistency of LLM’s responses across sequential examination attempts. .

LLM	Outcome	1^st^ to 2^nd^ attempt	2^nd^ to 3^rd^ attempt	3^rd^ to 4^th^ attempt	4^th^ to 5^th^ attempt
GPT-3.5	No change in response	255 (76%)	264 (79%)	254 (76%)	224 (67%)
	Incorrect to correct response	20 (6%)	25 (7%)	20 (6%)	24 (7%)
	Correct to incorrect response	29 (9%)	18 (5%)	23 (7%)	19 (6%)
	Incorrect to incorrect response	31 (9%)	28 (8%)	38 (11%)	38 (11%)
GPT-4	No change in response	305 (91%)	300 (89%)	298 (89%)	303 (90%)
	Incorrect to correct response	13 (4%)	12 (4%)	13 (4%)	12 (4%)
	Correct to incorrect response	10 (3%)	14 (4%)	18 (5%)	11 (3%)
	Incorrect to incorrect response	7 (2%)	9 (3%)	6 (2%)	9 (3%)
Llama-2	No change in response	300 (90%)	310 (92%)	304 (91%)	308 (92%)
	Incorrect to correct response	20 (2%)	25 (3%)	20 (2%)	24 (2%)
	Correct to incorrect response	29 (2%)	18 (1%)	23 (4%)	19 (2%)
	Incorrect to incorrect response	31 (6%)	28 (4%)	38 (3%)	38 (4%)

**Figure 5:**
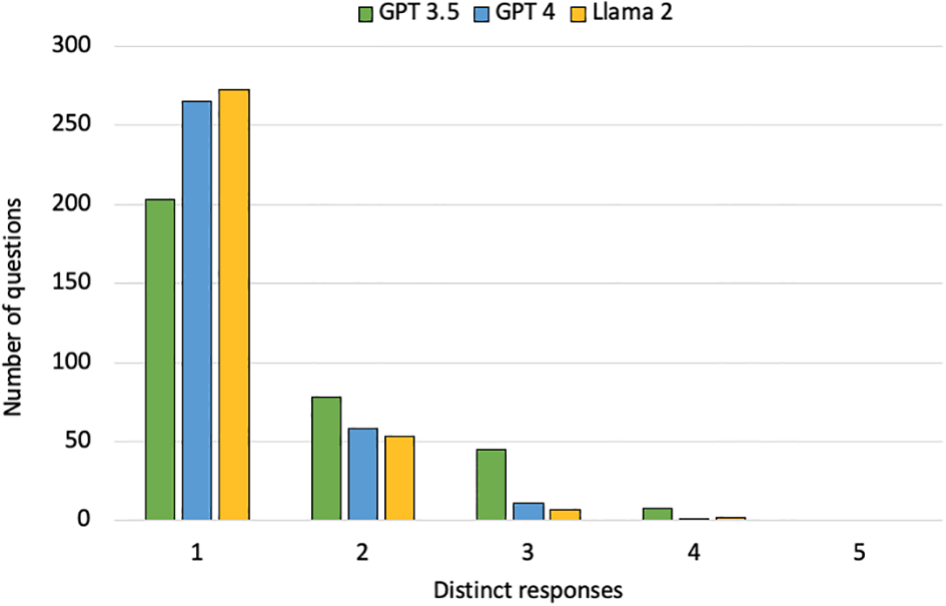
The number of distinct answers generated by each LLM when each model was queried with the same set of 335 questions, each repeated five times.

GPT-3.5 provided identical answers across all five attempts for 203 of 335 questions (61%), while GPT-4 and Llama 2 demonstrated higher consistency, providing consistent answers for 265 (79%) and 273 (81%) questions, respectively. GPT-3.5 generated two different answers for 78 questions (23%), compared to 58 questions (17%) for GPT-4 and 53 questions (16%) for Llama 2. The models produced three different answers with de-creasing frequency: GPT-3.5 for 45 questions (13%), GPT-4 for 11 (3%), and Llama 2 for 7 (2%). Instances of four different answers in response to the same question were rare: GPT-3.5 in 8 cases (2%), GPT-4 in 1 case (<1%), and Llama 2 in 2 cases (<1%) (**Figure 5**).

### 3.3 Response length and detail generated by large language models

**Figure 6:**
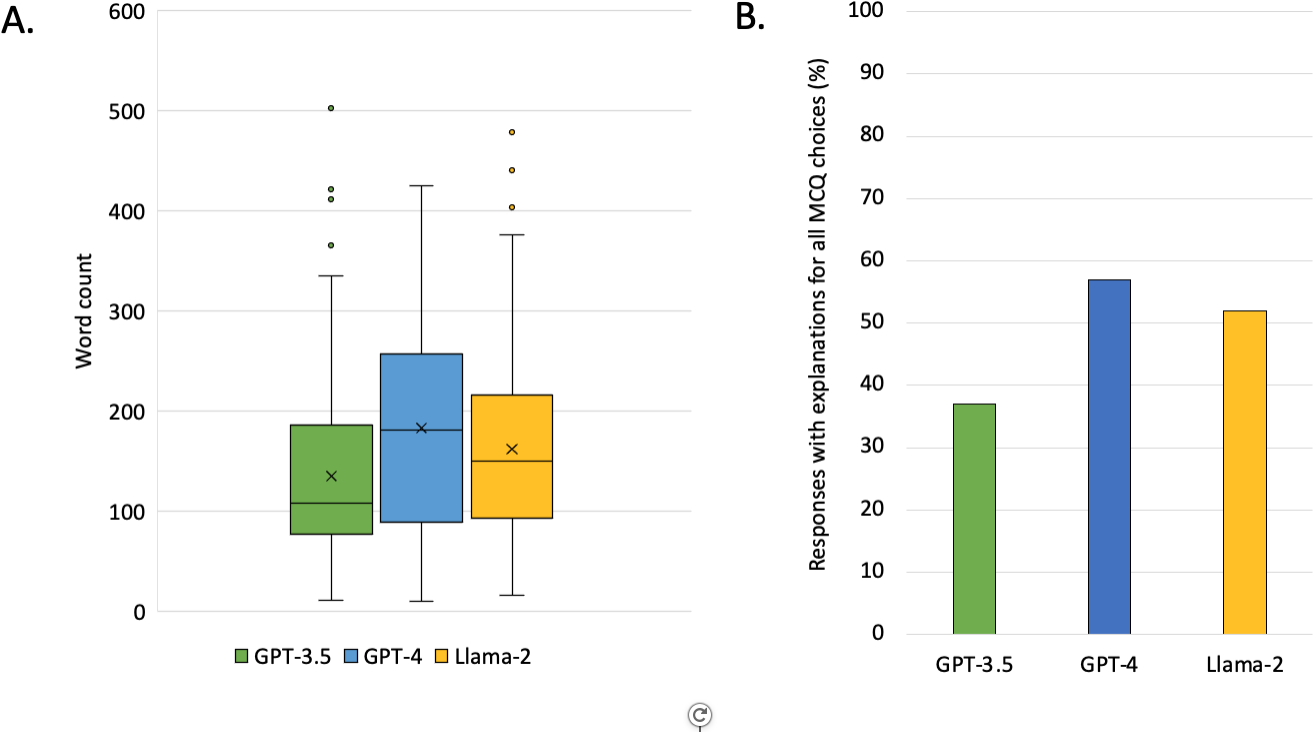
**(A)** Box plot representing the average word count of outputs generated by each LLM. **(B)** Frequency of comprehensive answer explanations by large language model.

The level of detail in responses generated by each LLM varied substantially. The average word count of response explanations was 134 words for GPT-3.5, 183 words for GPT-4, and 162 words for Llama-2 (**Figure 6A**). In response to multiple-choice questions, all three LLMs frequently provided explanations not only for why a selected option was correct, but also for why the remaining answer options were incorrect, despite no explicit prompting to do so. GPT-3.5 provided detailed rationales addressing all answer choices in 124 of 335 questions (37%), compared with 191 of 335 questions (57%) for GPT-4 and 173 of 335 questions (52%) for Llama-2 (**Figure 6B**).

## 4 Discussion

LLMs are advanced artificial intelligence systems which use transformer-based architectures with self-attention mechanisms to better understand context and semantics compared to traditional neural networks (1). Trained on massive datasets including books, articles, and websites, they learn linguistic patterns during pre-training, enabling them to generate human-like text across various domains. Models such as LLaMA 65B and ChatGPT-3 are trained on trillions of tokens (20, 21), making them valuable tools in fields like healthcare, software development, and education. In this study, we evaluated the performance of three widely used large language models—GPT-3.5, GPT-4, and Llama-2—on a domain-specific radiation and cancer biology examination using a structured benchmarking framework. This approach provides a standardized method for evaluating LLM knowledge within a defined domain while minimizing the risk of data leakage and enabling more meaningful comparisons across models and timepoints.

To evaluate the performance of these LLMs, we used the 2023 ASTRO Radiation and Cancer Biology Exam Study Guide. This study guide is produced by the ASTRO Radiation and Cancer Biology Study Guide Task Force to assist Radiation Oncology residents in preparation for the Radiation and Cancer Biology component of the computer-based Qualifying Exam. Previous years’ exams were not used as at the time of data collection, the LLMs were trained on data up until September 2021, therefore only the 2023 ASTRO exam represented out-of-training-data. A total of 335 MCQ questions were queried by each LLM. GPT-3.5 scored 221/335 (62%), GPT-4 scored 271/335 (81%), and Llama-2 scored 175/335 (51%). Although the qualifying exam’s pass/fail rate is determined by applying a criterion-referenced exam standard, thus there is no set pass/fail rate, anecdotes from staff oncologists who have written the exam estimate that a score above 50% is likely a pass and a score of 81% is a good score.

Our findings show that the three LLMs studied have varied levels of knowledge and ability to interpret questions related to cancer and radiation biology. GPT-4 significantly outperformed GPT-3.5 and Llama-2. GPT-3.5 also significantly outperformed Llama-2. Potential reasons for GPT-4’s superior accuracy may be its extensive training data, model architecture, or fine-tuning strategies (22). These results are in keeping with other studies comparing the performance of multiple LLMs on medical and oncology related examination questions (16, 23–25). Score discrepancies across different foundational domains may be due to data availability, as well as quality and quantity of training datasets.

It should be noted that all LLMs assessed in our study showed markedly worse performance in questions requiring the use of calculation(s); for instance, questions related to linear energy transfer, cell survival curves, cell cycle kinetics, and dose fractionation schedules. It has previously been shown that LLMs have limited performance when solving arithmetic reasoning and calculation tasks (26). Unlike natural language understanding, calculations typically have a single correct answer, making the task of generating accurate solutions more challenging. Moreover, they require specific abstraction and reasoning skills that are not well supported by the architecture and training of language models.

A key consideration in the use of LLMs in medical applications is their reliability, which refers to the models’ ability to provide consistent answers to identical prompts when tested multiple times (18). Our study found notable inconsistencies in the responses across five evaluation sessions, where GPT-3.5 exhibited greater variability than GPT-4 and Llama-2. To further characterize response variability, we analyzed transitions between consecutive evaluation runs, capturing patterns such as consistent responses, improvement (incorrect to correct), degradation (correct to incorrect), and persistent error. Sequential comparisons were selected to reflect how model outputs may vary across repeated use over time. In addition, we examined the total number of distinct responses generated per question across all trials, providing a complementary measure of global variability. Together, these analyses demonstrate that even when overall accuracy remains stable, individual responses may fluctuate, which has important implications for reliability in educational and clinical contexts.

Such variability can be attributed to several factors including the inherent non-deterministic nature of LLMs (18, 27), meaning the same input can yield different results, some of which may be incorrect or “hallucinated”. This variability is often due to the probabilistic nature of token selection during generation. These findings align with previous research that highlighted similar inconsistencies in LLMs’ responses (6, 28, 29).

When interpreting incorrect outputs generated by LLMs, it is important to distinguish between different underlying failure modes. In some cases, models may consistently produce the same incorrect response, potentially reflecting systematic knowledge gaps or inaccuracies in the training data. In other cases, incorrect responses may arise stochastically from probabilistic generation processes, commonly referred to as hallucinations (30). Although hallucinations can be formally characterized using structured error taxonomies (31) or entropy-based uncertainty estimators (30), such analyses were beyond the scope of the present study.

Despite the absence of formal hallucination quantification, qualitative review of incorrect responses revealed that most erroneous answers were accompanied by detailed and plausible explanations. We observed both variability, in which different incorrect answers were generated across repeated trials for the same question, and consistency, in which the same incorrect answer was repeatedly produced. Together, these patterns suggest that both stochastic hallucination and systematic deficiencies in model knowledge contributed to incorrect responses, underscoring the need for human oversight and continued refinement of LLMs prior to their use in educational or clinical settings.

Across models, GPT-3.5 demonstrated moderate accuracy but substantial response variability, whereas GPT-4 achieved higher accuracy with greater consistency across examination attempts. Llama-2 showed lower overall accuracy but comparatively high consistency. Notably, all three LLMs generated detailed explanations without explicit prompting, often addressing both correct and incorrect answer options. GPT-4 was the most verbose, with the highest average word count per response. However, this verbosity represents a double-edged sword: while detailed explanations may enhance perceived usefulness, they may also amplify the risk of convincingly presented misinformation. In technical domains such as radiation and cancer biology, such fluent but incorrect explanations may be particularly misleading to non-expert users (9).

An important methodological consideration in this study is the use of a closed-book, no-prompt design, in which models were evaluated without additional instruction or access to external information sources. This approach was intentionally chosen to isolate intrinsic model knowledge and enable standardized comparisons across systems. However, it differs from real-world applications, where users often employ prompt engineering techniques or retrieval-augmented generation (RAG) to enhance performance (32). As a result, the accuracy reported in this study likely underestimates achievable performance in practical settings. Nonetheless, baseline evaluation remains important for identifying fundamental knowledge gaps, limitations in reasoning—particularly for calculation-based questions—and inherent variability in model outputs.

Several limitations of this study warrant consideration. First, the use of a single-year, single-source question set limits generalizability and may not fully represent the breadth of radiation oncology knowledge or clinical decision-making. Second, the exclusion of image-based questions restricts evaluation of multimodal reasoning, which is increasingly relevant as newer models incorporate visual inputs. Third, the absence of a human comparator prevents direct benchmarking against trainee or expert performance, and thus this study should not be interpreted as a validation of clinical competence. Rather, the ASTRO study guide serves as a standardized and specialty-specific knowledge assessment tool, appropriate for benchmarking but not a surrogate for real-world clinical expertise. Finally, as large language models continue to evolve rapidly, the results presented here reflect model capabilities at a specific timepoint and may not generalize to newer model versions, emphasizing the continuous need for reassessment.

Further research on the application of LLMs in Radiation Oncology is required to improve our understanding of their potential and limitations. This includes exploring a broader array of AI models, particularly those that are trained in radiation oncology or capable of interpreting complex medical imaging. Going forward, we expect large language models will be benchmarked against expert clinicians rather than solely against standardized examinations, representing a fundamental shift in how AI performance is evaluated in medicine (33). Longitudinal studies would also be valuable in tracking the progress and adaptation of LLMs over time, shedding light on their learning curves, improvements in accuracy, reliability, and ability to integrate new data.

## 5 Conclusions

Using the 2023 ASTRO Radiation and Cancer Biology practice examination, this study provides a methodological benchmark of large language model performance on domain-specific, out-of-training-data content relevant to radiation oncology education. Although differences in accuracy were observed among models, all LLMs exhibited important limitations, including reduced performance on calculation-based questions and response variability across repeated evaluations. These findings underscore the value of standardized, out-of-training-data benchmarking frameworks and the need for cautious interpretation of LLM outputs in specialized educational contexts.

## Data Availability

The datasets presented in this study can be found in online repositories. The names of the repository/repositories and accession number(s) can be found below: 10.6084/m9.figshare.29555648.
